# Gene Expression Profiling of Multiple Histone Deacetylases (*HDAC*) and Its Correlation with *NRF2*-Mediated Redox Regulation in the Pathogenesis of Diabetic Foot Ulcers

**DOI:** 10.3390/biom10101466

**Published:** 2020-10-21

**Authors:** Rajan Teena, Umapathy Dhamodharan, Daoud Ali, Kesavan Rajesh, Kunka Mohanram Ramkumar

**Affiliations:** 1 Department of Biotechnology and SRM Research Institute, SRM Institute of Science and Technology, Kattankulathur, Tamil Nadu 603 203, India; teena.rajan3@gmail.com (R.T.); usadhamodharan@gmail.com (U.D.); 2 Department of Zoology, College of Science King Saud University, P.O. Box 2455, Riyadh 11451, Saudi Arabia; aalidaoud@ksu.edu.sa; 3 Department of Podiatry, Hycare Super Speciality Hospital, MMDA Colony, Arumbakkam, Chennai, Tamil Nadu 603 203, India

**Keywords:** Nrf2, epigenetics, HDACs, sirtuins, angiogenesis, diabetic wounds

## Abstract

Nuclear factor erythroid-2-related factor 2 (Nrf2) is a protein of the leucine zipper family, which mitigates inflammation and employs cytoprotective effects. Attempting to unravel the epigenetic regulation of type 2 diabetes mellitus (T2DM) and diabetic foot ulcer (DFU), we profiled the expression of eleven isoform-specific histone deacetylases (*HDACs*) and correlated them with *NRF2* and cytokines. This study recruited a total of 60 subjects and categorized into DFU patients (*n* = 20), T2DM patients (*n* = 20), and healthy controls (*n* = 20). The DFU patients were subcategorized into uninfected and infected DFU (*n* = 10 each). We observed a progressive decline in the expression of *NRF2* and its downstream targets among T2DM and DFU subjects. The inflammatory markers *IL-6* and *TNF-α* were significantly upregulated, whereas anti-inflammatory marker *IL-10* was significantly downregulated in DFU. Of note, a significant upregulation of *HDAC1, 3, 4, 11, SIRT3* and downregulation of *HDAC2,8, SIRT1, SIRT2, SIRT3, SIRT7* among DFU patients were observed. The significant positive correlation between *NRF2* and *SIRT1* in DFU patients suggested the vital role of *NRF2/SIRT1* in redox homeostasis and angiogenesis. In contrast, the significant negative correlation between *NRF2* and *HDAC1*, *3* and *4*, implied an imbalance in *NRF2-HDAC1, 3, 4* circuit. Furthermore, a significant positive correlation was observed between *HDAC4* and *IL-6,* and the negative correlation between *SIRT1* and *IL-6* suggested the pro-inflammatory role of *HDAC4* and the anti-inflammatory role of *SIRT1* in *NRF2* signaling. In conclusion, the epigenetic changes such as upregulation of *HDAC1, 3, 4, 11, SIRT3* and downregulation of *HDAC2, 8, SIRT1, SIRT2, SIRT6, SIRT7* and their association with *NRF2* as well as inflammatory markers are suggestive of their roles in pathophysiology of T2DM and DFU.

## 1. Introduction

Diabetic foot ulcer (DFU) is an extremely prevalent complication of diabetes mellitus that causes ulcers in the lower limbs of the affected individuals. If not treated properly, these ulcers become infected and severely degrade the skin tissue and the bones, leading to lower-extremity amputations. There are diverse risk factors that contribute to its pathogenesis. Among these, the most critical factors include poor glycemic control, inflammation, oxidative stress, peripheral neuropathy, autonomic neuropathy, and micro and macroangiopathy [1]. Management of DFU involves multidisciplinary and integrative approaches such as anti-microbial dressing materials, surgical debridement, specialized footwear, hydrogel-based dressing materials, oxygen therapies such as hyperbaric oxygen therapy (HBOT) and ozone therapy, vacuum-assisted wound closure, revascularization procedures, etc. [2]. It is widely accepted that treatment strategies that combat cellular oxidative stress bolster and accelerate the wound healing process [3]. One of the crucial transcription factors that combat cellular oxidative stress is nuclear factor erythroid-2-related factor 2 (NRF2), and it is encoded by the gene *NRF2*. [4]. It transcribes antioxidant and detoxifying genes such as catalase (*CAT*), NAD(P)H quinone oxidoreductase-1 (*NQO1*), heme oxygenase-1 (*HO-1*), glutathione peroxidase (*GPx*), and superoxide dismutase (*SOD*) involved in cytoprotection. Hence, NRF2 is recognized to be the prime transcriptional regulator of redox homeostasis. However, in several diseases, including diabetes, the level of NRF2 is reported to be very low [5]. A recent study from our laboratory has provided insight into the role of HBOT in restoring NRF2 levels and angiogenesis in DFU subjects [6]. However, the cellular mechanisms that downregulate NRF2 in T2DM and DFU are still unclear.

Accumulating evidence demonstrates that genetic, as well as epigenetic factors, regulate *NRF2* expression [7,8]. Unlike genetic factors, epigenetic modifications happen gradually and alter the entire epigenome. Hence, epigenetic mechanisms play a pivotal role in maintaining chromatin structure and gene regulation [9]. The fundamental mechanisms of epigenetic alterations include DNA methylation, non-coding RNAs, histone variants, and their modifications. The epigenetic changes are primarily mediated by proteins that induce, eliminate, or discern covalent modifications to DNA or protein [10]. Hence, the abnormal regulation of these proteins leads to various diseases such as diabetes, neurodegenerative diseases such as Alzheimer’s disease, Parkinson’s disease, leukemia, and autoimmune diseases [11]. Among these regulatory proteins, the epigenetic “erasers” known as histone deacetylases (HDACs) have gained attention in the recent past due to their involvement in several disease states. Studies demonstrate that methylation of the CpG islands (CGIs) in a gene promoter recruits HDACs and causes gene silencing by erasing acetyl moieties from the lysine residues of histone and non-histone proteins, thereby inducing conformational changes in chromatin [12,13]. Based on yeast sequence homology and reaction mechanisms, the HDACs are classified into four classes, namely class-I (HDAC 1, 2, 3 and 8), class-II (HDAC 4, 5, 6, 7 and 9), class-III (SIRT 1–7), and class-IV (HDAC11).

Recently, Kang et al. investigated the epigenetic regulation of *NRF2* by DNA methylation by demonstrating 5-fluorouracil-induced oxidative stress resulted in the activation of ten–eleven translocation (TET) enzymes, hypomethylation of *NRF2* promoter, and induction of NRF2 activity, thereby enabling chemoresistance [14]. Although a few studies have provided insights on epigenetic regulation of *NRF2* by DNA methylation [15,16], the significance of other epigenetic modifications that regulate *NRF2*, especially the importance of *HDACs* in regulating *NRF2* expression, is yet to be established.

So far, the role of HDACs in several diseases has been investigated using preclinical studies [17]. These studies suggest that functions of certain HDACs are beneficial, whereas some have detrimental effects. To mention, among class II HDACs, HDAC4 has been reported to cause podocyte injury in diabetic nephropathy [18]. Intriguingly, it is also essential for the p53-dependent arrest of cancer [19,20,21]. The disparity in HDAC4 expression in diabetic nephropathy and cancer suggests its disease-dependent nature. It is possible that in diabetic nephropathy, activation of HDAC4 activates p53, thereby inhibiting NRF2 and consequently inducing podocyte injury. However, in cancer, this would help in the decline of NRF2 and the prevention of tumorigenesis [22].

Since different HDACs have diverse roles in various metabolic pathways, the concept of inhibition of HDACs using pan-HDAC inhibitors, that are known to inhibit both nuclear and cytoplasmic HDACs, may not accomplish without side effects. Hence, it is essential to decipher the functions of each HDACs.

The present study evaluated the gene expression profile of multiple histone deacetylases in the peripheral blood mononuclear cells (PBMCs) of a clinically well-characterized patient cohort comprising T2DM and DFU subjects, as PBMCs are surrogate tissues that have the property to mimic the human in vivo conditions. Further, the gene expression of HDACs was correlated with *NRF2* and inflammatory markers to identify the interplay of HDACs in redox control, angiogenesis, and pro-inflammation.

## 2. Materials and Methods

### 2.1. Study Subjects

Study subjects were recruited from Hycare Super Speciality Hospital, Chennai. They were categorized into three groups—the group I: subjects with normal glucose tolerance (NGT, *n* = 20), group II: subjects with type 2 diabetes mellitus (T2DM, *n* = 20), group III: subjects with DFU (*n* = 20). NGT comprised of healthy subjects with fasting plasma glucose (FPG) < 100 mg/dL and 2 h postprandial plasma glucose (PPG) ≤ 140 mg/dL during an oral glucose tolerance test. T2DM comprised of subjects with FPG level of ≥ 126 mg/dL and/or PPG level of ≥ 200 mg/dL [23]. DFU subjects were subcategorized into group IIIa: subjects with uninfected DFU (UI-DFU, *n* = 10) and group IIIb: subjects with infected DFU (I-DFU, *n* = 10) based on Infectious Diseases Society of America (IDSA) and the International Working Group on the Diabetic Foot (IWGDF) guidelines on the classification of DFU. Wounds without purulence or appearances of inflammation were categorized as uninfected (grade 1). Wounds with the occurrence of two or more appearances of inflammation, purulent discharge, lymphangitis, erythema >2 cm, osteomyelitis, white blood cells (WBC) count (>12,000 or <4000 cells/microliter or ≥10 % immature cells) were categorized as infected (grade > 2) [24]. Subjects with auto-immune disorders, gestational diabetes, cardiovascular diseases, and inflammatory, infectious, rheumatic, and hematological disorders were excluded. Informed consent was obtained from the participants of the study, and the blood samples were collected in the fasting state. A comprehensive quality management practice ensured that the samples are of quality and a suitable fit for the proposed investigations. The study protocol was permitted by the Institutional Ethics Clearance committee (025-A/HYC/IEC/2018) and was carried out with the guidelines of the Declaration of Helsinki.

### 2.2. Basic Clinical and Biochemical Characteristics of the Study Participants

Standard protocols were followed to record the anthropometric measurements and the blood pressure of the participants. Medical history was obtained from all the participants, including details of their occupation, severity and duration of the disease, treatment protocol followed, complications encountered, and addiction, if any. The systolic blood pressure (SBP) and diastolic blood pressure (DBP) were measured using INFI deluxe mercury sphygmomanometer. The plasma glucose levels in both fasting (FPG) and postprandial (PPG) state were analyzed by the standard protocol. Glycated hemoglobin (HbA1c) levels were analyzed using HPLC (Bio-Rad, Hercules, CA). The levels of total serum cholesterol (TSC), high-density lipoprotein cholesterol (HDL-c), low-density lipoprotein cholesterol (LDL-c) and creatinine were measured using standard protocols. Homeostatic model assessment of insulin resistance (HOMA-IR) was analyzed as described previously [25]. C-reactive protein (CRP) was evaluated using Randox Daytona analyzer (BioAgilytix, Durham, NC, USA). WBC counts were analyzed on a hematology analyzer (XN-1000, Japan). Vibration perception threshold (VPT) was adopted to identifying distal symmetrical peripheral neuropathy using biothesiometer (Bio-medical Instruments Co., Newbury, OH, USA). The study participants with a VPT above 25V were considered neuropathy patients, and those with 16–24 V were considered as susceptible to neuropathy [26]. Ankle-brachial index (ABI), a non-invasive tool, was adopted to assess the vascular status, and ABI ≤ 0.9 was considered as having PVD [27].

### 2.3. Sample Size Calculation and Power of the Study

A pilot study was conducted with ten subjects per group. Based on the results, with a 95% confidence interval (CI), an estimated *p*-value of < 0.05 and a power of 80%, the present sample size was derived.

### 2.4. Isolation of Peripheral Blood Mononuclear Cells (PBMCs) from Blood

Four to five ml of venous blood was collected from the study subjects in heparinized vacutainers. It was gently layered on the top four milliliters of ficoll histopaque 1077 (Sigma Aldrich, St. Louis, MO, USA) and centrifuged for thirty minutes at 5000 rpm in four degrees Celsius without a break. The white buffy coat (PBMCs) formed in the interphase of plasma and ficoll histopaque was carefully aspirated. Further, it was suspended in PBS and centrifuged at 10,000 rpm, and the pellet was incubated in the ammonium-chloride-potassium lysing buffer (Thermo Fisher Scientific, Waltham, MA, USA) for ten minutes and was rinsed with PBS. The isolated PBMCs were used for expression studies.

### 2.5. Quantitative RT-PCR Analysis

RNA was isolated using the RNeasy Mini Kit (Qiagen) according to the kit’s protocol. The concentration of RNA was estimated using NanoDrop™ 2000/2000c Spectrophotometer (Thermo Fisher Scientific, Waltham, MA, USA). RNA with a purity of 2.0 was used for cDNA conversion. Briefly, 1 µg of RNA was mixed with Takara PrimeScript^TM^ RT reagent kit components, namely, PrimeScript RT Enzyme Mix I, 5X PrimeScript buffer, Oligo dT primers, random 6-mers and RNase free water-based on manufacturer’s instructions. Further, the reaction mix was incubated in Bio-Rad S1000 thermal cycler. The amplification protocol consists of first-strand cDNA synthesis (42 °C; 15 min) and enzyme deactivation (85 °C; 30 s). The resultant cDNA samples with a purity of 1.8 were used for quantitative RT-PCR analysis. Quantitative RT-PCR was carried out using CFX Connect Real-Time PCR System (Bio-Rad, Hercules, CA, USA). The reaction mixture consisted of SYBR^®^ Premix ex taq™ II (Clontech, Takara, Japan), 10 µM primers, and 500 ng cDNA made up to 12.5 µL nuclease-free water. Each reaction was performed in triplicates to improve reliability and the average Cq value was used for quantification and analysis. For each experiment, non-template control was carried out to avoid false-positive results. The primers used for the experiment are listed in Table 1. The expression of target genes was calculated using the formula 2^−ΔΔCt^ and was normalized to the housekeeping gene glyceraldehyde 3-phosphate dehydrogenase (*GAPDH*).

### 2.6. Statistical Analysis

The clinical and biochemical characteristics of the study subjects are expressed as mean ± SD. The Mann–Whitney U test was carried out to compute the statistical significance. Spearman’s correlation was performed to analyze the correlation of *HDACs* with *NRF2* and inflammatory markers. *p* values < 0.05 were considered statistically significant. All the statistical analysis was carried out using SPSS version 20.0 and GraphPad Prism 8.4.2.

## 3. Results

### 3.1. Clinical and Biochemical Characteristics of the Study Subjects

Table 2 depicts the clinical and biochemical characteristics of the study participants. The NGT and T2DM group’s mean age was 51.6 ± 1.3 and 51.5 ± 1.2 years, respectively. For uninfected and infected DFU subjects, it was 51.7 ± 1.2 and 51.5 ± 1.3 years, respectively. SBP, DBP, FPG, PPG, HbA1c, TSC, LDL-c, HOMA-IR, urea, creatinine, CRP, ESR, and WBC counts were observed to be significantly elevated in T2DM subjects when compared with NGT subjects. In contrast, HDL-c did not show any significant difference. Besides, infected DFU subjects had a significant increase in SBP, DBP, FPG, PPG, HbA1c, LDL-c, HOMA-IR, urea, CRP, ESR, and WBC counts when compared with uninfected DFU subjects. However, BMI, TSC, HDL-c, and creatinine did not show any significant difference.

### 3.2. Expression of NRF2 and Downstream Targets in PBMCs

**Figure 1:**
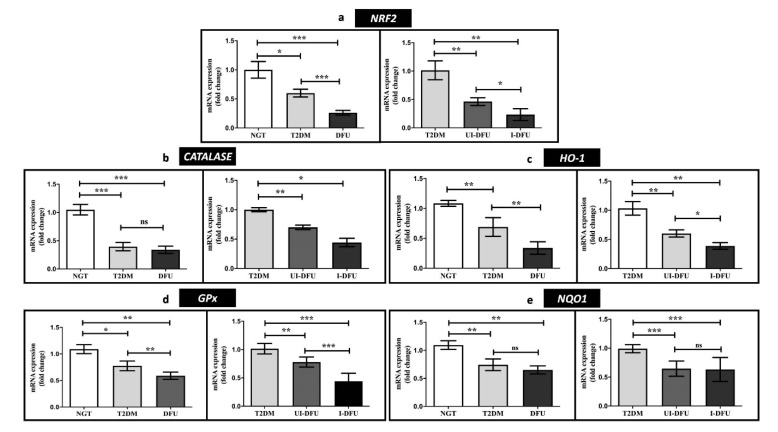
Relative gene expression of (**a**) *NRF2* (**b**) *CAT* (**c**) *HO-1* (**d**) *GPx*, and (**e**) *NQO1* in PBMCs of study subjects analyzed using qPCR. Data are represented as mean ± SEM. * *p* < 0.05; ** *p* < 0.01; *** *p* < 0.001, and ^ns^ nonsignificant.

The expression of the transcription factor, *NRF2* (3.8-fold, *p* < 0.001) (Figure 1a) and downstream targets such as *CAT* (2.5-fold, *p* < 0.001) (Figure 1b), *HO-1* (3-fold, *p* < 0.001) (Figure 1c), *GPx* (1.83-fold, *p* < 0.001) (Figure 1d), and *NQO1* (1.69-fold, *p* < 0.001) (Figure 1e) were significantly decreased in DFU subjects with respect to NGT subjects. Besides, these were observed to be least expressed in the infected DFU subjects when compared to the T2DM subjects.

### 3.3. Quantitative RT-PCR Analysis of HDACs

**Figure 2:**
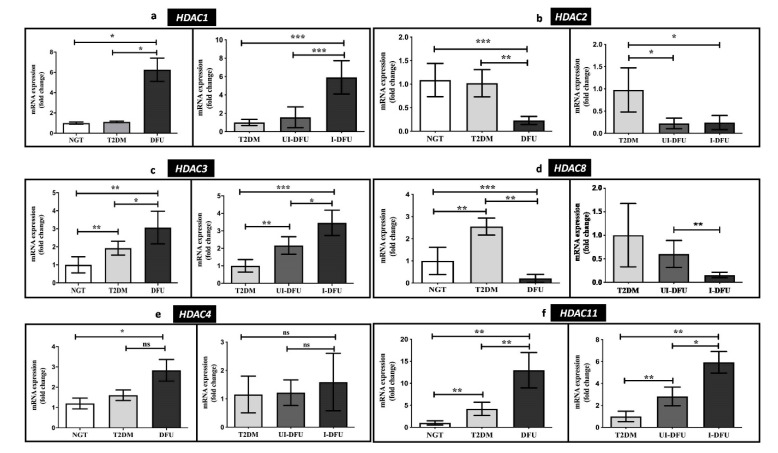
Relative gene expression of (**a**) *HDAC1* (**b**) *HDAC2* (**c**) *HDAC3* (**d**) *HDAC8* (**e**) *HDAC4*, and (**f**) *HDAC11* in PBMCs of the study subjects measured using qPCR. Data are represented as mean ± SEM. * *p* < 0.05; ** *p* < 0.01; *** *p* < 0.001, and ^ns^ nonsignificant.

The expression of class-I *HDACs* namely *HDAC 1*, *2*, *3*, and *8* was analyzed in the PBMCs of the study population. The results are depicted in Figure 2a–d. *HDAC1* (6-fold, *p* < 0.05) and *HDAC3* (3-fold, *p* < 0.01) were significantly increased in DFU when compared to the NGT. In particular, a concomitant increase in *HDAC1* (6-fold, *p* < 0.001) and *HDAC3* (3-fold, *p* < 0.001) were seen among the infected DFU subjects when compared to the T2DM. On the other hand, *HDAC2* (9-fold, *p* < 0.001) was significantly decreased in DFU when compared to the NGT, and its expression was progressively downregulated in the uninfected (1-fold, *p* < 0.05) and infected DFU subjects (1.5-fold, *p* < 0.05) when compared to the T2DM. Our analysis also revealed that *HDAC8* was significantly upregulated in T2DM (2.5-fold, *p* < 0.01) and significantly downregulated in the DFU (4.7-fold, *p* < 0.001). Besides, *HDAC8* was significantly low among the infected DFU subjects (6-fold, *p* < 0.01). Furthermore, the gene expression of *HDAC4* was significantly increased among the DFU subjects (2-fold, *p* < 0.05) when compared to NGT (Figure 2e). As depicted in Figure 2f, the expression of *HDAC11,* the class-IV HDAC was significantly upregulated in T2DM (4-fold, *p* < 0.01) and DFU (11-fold, *p* < 0.01). In addition, it was significantly upregulated in both infected (6-fold, *p* < 0.01) and uninfected DFU (2.8-fold, *p* < 0.01) subjects when compared to the T2DM.

**Figure 3:**
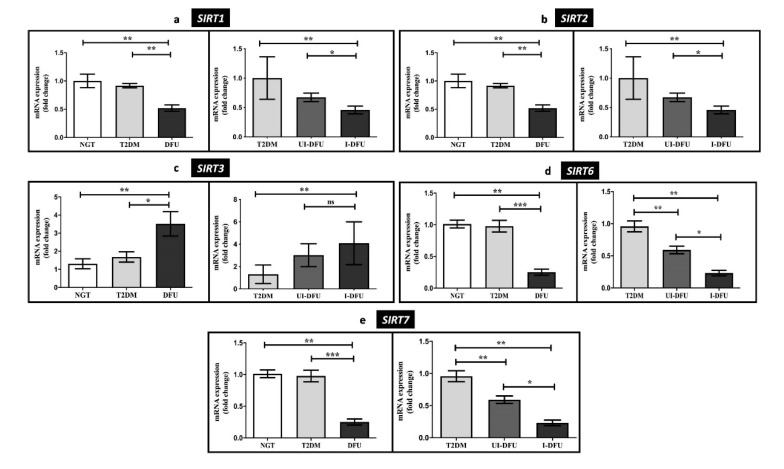
Relative gene expression of (**a**) *SIRT1* (**b**) *SIRT2* (**c**) *SIRT3* (**d**) *SIRT6*, and (**e**) *SIRT7* measured in PBMCs of the study subjects by qPCR. Data are represented as mean ± SEM. * *p* < 0.05; ** *p* < 0.01; *** *p* < 0.001, and ^ns^ nonsignificant.

The class-III HDACs, *SIRT1, 2, 3, 6*, *7* were analyzed, and the results are represented in Figure 3a–e. The sirtuins, namely, *SIRT1* (2-fold, *p* < 0.01), *SIRT2* (2-fold, *p* < 0.01), *SIRT6* (2-fold, *p* < 0.001), and *SIRT7* (4-fold, *p* < 0.001), were significantly decreased in DFU when compared to the NGT. In addition, there was a significant decrease in these sirtuins among infected DFU subjects when compared to uninfected DFU subjects. In contrast, *SIRT3* alone was significantly enhanced in DFU (3.5-fold, *p* < 0.01) compared to T2DM and NGT. Besides, it was significantly elevated, particularly among the infected DFU subjects.

### 3.4. Transcriptional Levels of Pro-Inflammatory and Anti-Inflammatory Markers

**Figure 4:**
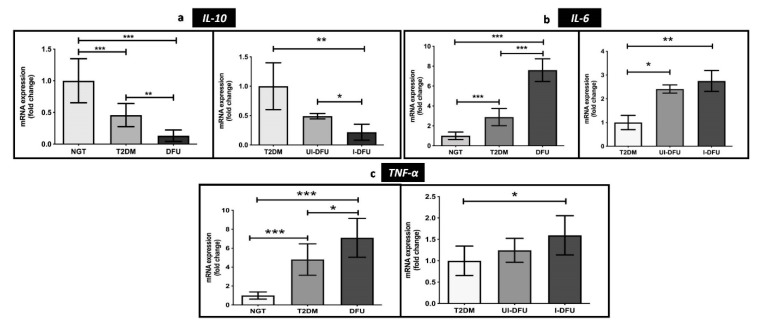
Relative gene expression of (**a**) *IL-10* (**b**) *IL-6*, and (**c**) *TNF-α* measured in PBMCs of the study subjects using qPCR. Data are represented as mean ± SEM. * *p* < 0.05; ** *p* < 0.01, and *** *p* < 0.001.

As represented in Figure 4a–c, the transcriptional levels of pro-inflammatory cytokines *IL-6* and *TNF-α* were significantly upregulated in T2DM (*IL-6*: 2.8-fold; *p* < 0.001; *TNF-α*: 8-fold; *p* < 0.001) and DFU (*IL-*6: 7-fold; *p* < 0.001; *TNF-*α: 7-fold; *p* < 0.001) when compared to the NGT.

**Figure 5:**
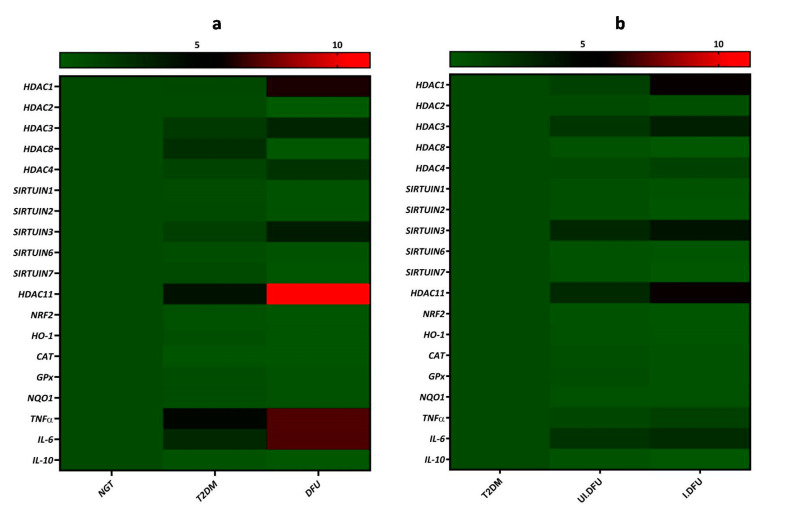
Heatmap showing the differential expression of *HDAC* isoforms, *NRF2*, and its downstream target genes and inflammatory cytokines in PBMCs of (**a**) T2DM and DFU subjects compared to NGT subjects (**b**) uninfected and infected DFU subjects when compared to T2DM subjects, created using GraphPad Prism 8.4.0.

Of note, we observed a significant upregulation of these cytokines among the infected DFU subjects when compared to the uninfected DFU subjects, indicating the inhibitory roles of interleukin 6 (*IL-6*) and tumor necrosis factor alpha (*TNF-α*) in wound healing. In contrast, anti-inflammatory cytokine interleukin 10 (*IL-10*) was significantly downregulated in DFU, particularly in the infected DFU subjects, when compared to the NGT. These indicate the crucial role of *IL-10* in mediating wound healing by suppressing inflammation. The heat map depicted in Figure 5 summarizes the differential expression of genes analyzed in the study.

### 3.5. Correlation of HDACs with NRF2 and Inflammatory Markers

**Figure 6:**
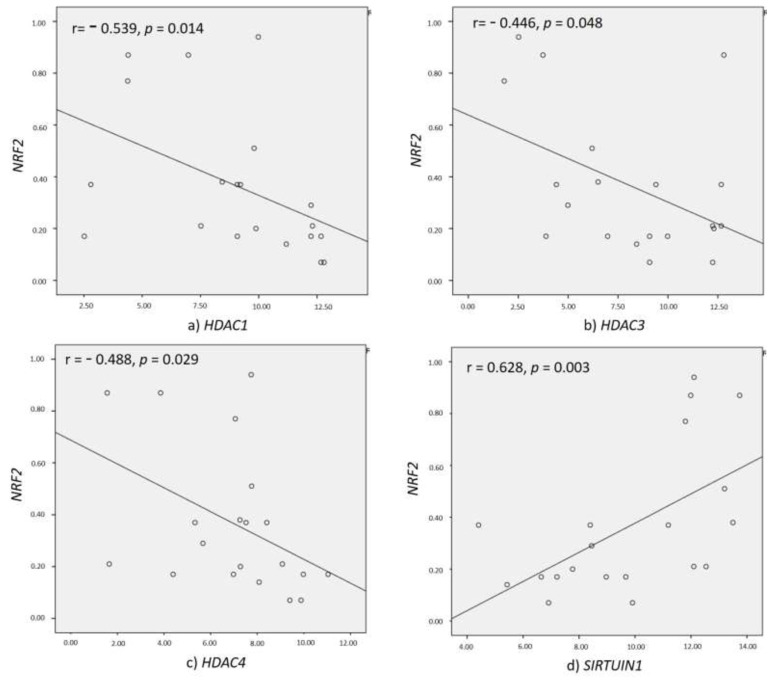
Spearman’s correlation coefficient of *NRF2* with epigenetic markers (**a**) *HDAC1* (**b**) *HDAC3* (**c**) *HDAC4*, and (**d**) *SIRT1* among DFU subjects. *p* and r values were calculated using the Spearman’s correlation test at 95% confidence intervals.

Table 3 shows the Spearman’s correlation of eleven isoforms of *HDACs* and *NRF2*. As depicted in Figure 6a–d, *NRF2* was positively correlated with *SIRT1* (r = 0.628, *p* = 0.003) and negatively correlated with *HDAC1* (r = −0.539, *p* = 0.014), *HDAC3* (r = −0.446, *p* = 0.048), and *HDAC4* (r = −0.488, *p* = 0.029). Table 4 shows the Spearman’s correlation of *HDACs* with inflammatory markers *IL-6*, *TNF-α* and *IL-10*. The *IL-6* expression showed a significant positive correlation with *HDAC4* (r = 0.733, *p* = 0.025) and a significant negative correlation with *SIRT1* (r = −0.683, *p* = 0.025).

## 4. Discussion

Under oxidative stress, NRF2 triggers cytoprotective genes and thereby offers cellular protection against electrophilic xenobiotics. Numerous studies have demonstrated the dysregulation of NRF2 in diabetes and several diseases [28,29]. Our laboratory has provided evidence on the dysregulation of NRF2 in T2DM patients [30]. In addition, we have demonstrated the pivotal role of *NRF2* in modulating *MALAT1/HIF-1α* loop essential for angiogenesis [31]. Similarly, Florczyk et al. have reported that the silencing of *NRF2* attenuates its angiogenic potential [32].

In the present investigation, we observed a progressive reduction in the expression of *NRF2* and its downstream target genes in PBMCs of T2DM and DFU patients. This suggests that decreased levels of *NRF2* could be a prime reason for impaired redox homeostasis and angiogenesis in the subjects with DFU. Hence, treatment strategies that improve *NRF2* can restore cellular homeostasis and promote diabetic wound healing.

The cellular mechanisms that dysregulate NRF2 are not well-explored. Hence, the present study sought to investigate the epigenetic signatures that dysregulate it. Dysregulation in epigenetic mechanisms leads to the pathogenesis of diabetes and associated complications [33]. Understanding HDAC expression is pivotal to understand the etiology of diabetes and foot ulcers. Several studies have reported that most of the CpG-rich regions in human gene promoters are unmethylated. However, in various malignancies, the CGIs in the transcriptional start site (TSS) were abnormally methylated. These sites recruit certain methyl CpG binding proteins with methylated DNA binding domains (MBD) to the DNA. These methyl CpG binding proteins form complexes with HDACs and chromatin remodeling proteins and thereby form inactive heterochromatin. In this way, HDACs play a crucial role in regulating gene expression [34].

A few reports have demonstrated that the application of a few HDAC inhibitors suppressed the dysregulation in HDACs. However, overexpression of all HDACs does not cause pathophysiology. Some play positive functions, including metabolic adaptations [35]. For example, SIRT1 can activate PGC-1α and stimulate FOXO1, thereby enhance mitochondrial function, insulin sensitivity, and thermogenic activity. [36].

Studies have demonstrated that the upregulation of class-I HDACs causes several malignancies and the inhibition of class-I HDACs aid in reducing insulin resistance. Johnson et al. have reported that class-I HDAC inhibitor romidepsin (FK228) reduced glucose levels in db/db mice [37]. Another study by Lkhagva et al. demonstrated that class-I and IIb HDAC inhibitor MPT0E014 reduces mitochondrial dysfunction and improves redox control in HL-1 cardiomyocytes [38]. In the present study, we observed a significant upregulation of *HDAC1* in the DFU subjects, particularly in the infected DFU subjects compared to the healthy subjects. Moreover, *HDAC1* was negatively correlated to *NRF2*. These suggest the possible role of *HDAC1* in suppressing wound healing and angiogenesis by downregulating NRF2. Hence, the regulation of diabetic wound healing by *NRF2/HDAC1* could be an efficient arena for therapeutic intervention.

Since HDAC1 and HDAC2 are part of a large deacetylase complex, they interact and deacetylate each other. Silencing of *HDAC1* increases *HDAC2* expression, and the silencing of *HDAC2* increases *HDAC1* expression [39]. Interestingly, a previous study by Nicolas et al. have evidenced that the siRNA-mediated knockdown of HDAC2 induces NRF2 instability, causing a deficiency in antioxidant expression in human bronchial epithelial cells [40]. Furthermore, transcriptional and translational downregulation of *HDAC2* has been noticed in surgically resected lung tissues of patients with severe chronic obstructive pulmonary disease [41]. In the present study, we observed a significant downregulation of *HDAC2* in the DFU subjects compared to T2DM and healthy controls. The reduction in *HDAC2* might be one of the factors that downregulate *NRF2* expression in DFU subjects and, consequently, wound healing.

Augmentation of HDAC3 leads to diabetes, and the present study showed its association with DFU [42]. Park et al. reported that knockdown of HDAC3 induces angiogenic VEGF and plasminogen activator inhibitor-1 [43]. A few studies also suggest that inhibition of HDAC3 using RGFP-966 in OVE26 diabetic mice with aortic pathologies activates NRF2 pathway by enhancing miRNA-200 expression [44]. A similar study evidenced that HDAC3 inhibitor RGFP-966 reduced T2DM induced blood–brain barrier permeability in diabetic mice by activating the NRF2 pathway [45]. The present study demonstrated that there is a significant increase in *HDAC3* expression among T2DM and DFU subjects. In addition, when compared to T2DM, both uninfected and infected DFU subjects showed a progressive augmentation in *HDAC3* expression. Moreover, *HDAC3* was inversely correlated to *NRF2*. These findings indicate the possible role of *HDAC3* in negatively regulating insulin resistance and angiogenesis by suppressing the NRF2 signaling cascade.

HDAC8, a unique class-I HDAC, recognizes both histone and non-histone substrates [46]. It is involved in the promotion of non-alcoholic fatty liver disease and hepatocellular carcinoma [47]. In cancer, HDAC8 is either deregulated or overexpressed and reported to interact with transcription factors [48,49]. Zhong et al. have demonstrated that poor glycemic control increased *HDAC8* in the retinal cells of streptozotocin (STZ)-induced diabetic rats by seventy to ninety percent compared to the normal age-matched rats [50]. These suggest the possible role of *HDAC8* in elevating insulin resistance and diabetes. In line with these findings, we observed a significant upregulation of *HDAC8* in T2DM patients. However, we noticed a remarkable decline in its expression among the uninfected and infected DFU subjects. A few studies suggest that HDAC8 plays a pivotal role in cell proliferation and that the inhibition of HDAC8 attenuates cell growth [51]. These suggest HDAC8 as a positive regulator of diabetic wound healing. Hence, the decline in HDAC8 might be one of the possible factors responsible for inefficient cell growth, proliferation, and angiogenesis in DFU. Since HDAC8 is low in DFU, an endogenous regulatory circuit of HDAC8 may delay wound healing in patients with DFU, and this needs to be researched in-depth in future investigations.

Recently, Wang et al. demonstrated the upregulation of class-II HDACs, namely, HDAC2,4,5, in STZ-induced diabetic rats, db/db mice, and kidney biopsies of diabetic subjects. Among these, the silencing of HDAC4 decreased podocyte injury by suppressing HDAC4-STAT1 signaling in STZ-induced diabetic rats [18]. HDAC4 is also reported to be involved in enhancing VCAM1 dependent vascular inflammation via activation of reactive oxygen species-dependent NFκB [52]. In the present investigation, we observed a significant increase in *HDAC4* expression among the DFU subjects. Moreover, *HDAC4* was also positively correlated to pro-inflammatory marker *IL-6* and inversely correlated to *NRF2*. These indicate that the elevation of *HDAC4* upregulates *IL-6*, suppressing wound healing, downregulating the NRF2 signaling pathway. The silencing of *HDAC4* would be a possible regulatory mechanism to enhance NRF2 levels in DFU subjects.

Inhibition of class-III HDACs can have negative impacts on metabolism [53]. Studies by Laemmle et al. have reported that inhibition of *SIRT1* in HCC cells suppressed *HIF1α* and its target gene *VEGF* responsible for angiogenesis [54]. Studies by Huang et al. have also demonstrated that Sirt1 and NRF2 form a positive feedback loop and inhibit diabetic nephropathy progression by decreasing fibronectin and TGF-β1 levels in glomerular mesangial cells (GMCs). Besides, Sirt1 is also known to activate NRF2 in GMCs treated with advanced glycation end products by deacetylating and reducing ubiquitination [55]. The present study demonstrated that the sirtuins *SIRT1*, *SIRT2*, *SIRT6,* and *SIRT7* are significantly declined in DFU subjects compared to T2DM and healthy controls. *NRF2* was positively-correlated with *SIRT1*, and *SIRT1* was negatively-correlated with *IL-6*. This suggests the vital role of *NRF2/SIRT1* in suppressing inflammation, enhancing redox homeostasis and angiogenesis. Thus, activation of *NRF2/SIRT1* pathway is an effective therapeutic strategy and will open new directions for diabetes and associated complications.

SIRT3 is a soluble mitochondrial matrix protein that regulates the enzymes involved in the rapid acetylation of multiple targets [56]. Studies on SIRT3-knockout (KO) mice suggest that SIRT3 aids in accelerating angiogenesis by ameliorating mitochondrial dysfunction [57,58]. However, in the present study, *SIRT3* was significantly elevated in DFU subjects when compared to NGT subjects. The difference in the results may plausibly be attributed to the study models. The present study used the PBMCs of clinically well-characterized T2DM and DFU subjects; in contrast, the aforementioned investigations were in animal models. However, our findings are in line with the data of Finley et al. which suggested that knockdown of SIRT3 in mouse embryonic fibroblasts (MEFs) is essential for enhanced HIF1α, glycolytic metabolism, and cellular proliferation [59]. Since *SIRT3* is over-expressed in DFU, downregulation of *SIRT3* via small molecule would serve as a potential therapy for diabetic wound healing.

Sun et al. have demonstrated that knockout of *HDAC11* in mice improved its resistance to metabolic syndrome and obesity by improving insulin sensitivity and glucose tolerance [60]. Studies by Bagchi et al. also proved that the deletion of HDAC11 inhibited the HDAC11/BRD2 association, exacerbated brown adipose tissue formation, and enhanced insulin sensitivity HDAC11-KO mice when fed with a high-fat diet. These findings suggest the function of *HDAC11* in regulating whole-body metabolism and demonstrate the significance of HDAC11 inhibition for the treatment of diabetes and its complications [61]. In the present investigation, we observed a significant increase in *HDAC11* among T2DM and DFU patients. Of note, there is an unusual increase in *HDAC11* expression among the uninfected and infected DFU patients compared to T2DM patients. These suggest the deleterious function of *HDAC11* in the pathogenesis of T2DM and DFU. It is also likely that *HDAC11* is one of the detrimental factors that impair *NRF2* expression among T2DM and DFU subjects. Collectively, the development of small molecules that suppress HDAC11 activity would be promising in treating diabetes and its associated complications.

## 5. Conclusions

Our findings demonstrate the dysregulation of the *NRF2* signaling cascade in T2DM and DFU and provide the first line of evidence on the expression profile of multiple HDAC isoforms in T2DM and DFU. It suggests that *NRF2* is inversely correlated with the *HDAC1, 3, 4* circuit and positively correlated with *SIRT1*. Furthermore, it also demonstrates that pro-inflammatory marker *IL-6* is positively correlated with *HDAC4* and negatively correlated with *SIRT1.* The *HDAC* expression profile and its association with *NRF2* as well as inflammatory markers, are suggestive of clinicopathological characteristics of patients with T2DM and DFU. These findings would support the development of HDAC inhibitors that are selective and isoform-specific. This would promote epigenetic reactivation of *NRF2* and be a promising therapeutic approach to ameliorate pathophysiological conditions in metabolic disorders.

## Figures and Tables

**Table 1:** List of primer sequences used for qPCR analysis.

Gene Names	Gene	Forward Primer	Reverse Primer
***NRF2 and downstream target genes***	*NRF2*	TGTAGATGACAATGAGGTTTC	ACTGAGCCTGATTAGTAGCAA
	*CAT*	ATCCGTGTAACCCGCTCATC	ACCTTCATTTTCCCCTGGGG
	*HO-1*	GGGAATTCTCTTGGCTGGCT	AACTGAGGATGCTGAAGGGC
	*GPx*	TATCGAGAATGTGGCGTCCC	CAAACTGGTTGCACGGGAAG
	*NQO1*	AGTCATCTCATTCCACTGTTGG	GCTGTCTCCCATTTTTCAGG
***HDACs***	*HDAC1*	GGCTGGCAAAGGCAAGTAT	CGCACTAGGCTGGAACATCT
	*HDAC2*	ATTGGGGAACAGGTGGTG	GGGGCGAGGGATAAAAGA
	*HDAC3*	GTATGAAGTCGGGGCAGAGA	CGTGGGTTGGTAGAAGTCC
	*HDAC8*	GTGGGAATTGGCAAGTGTCT	CCAGCACATAATCAGGACCA
	*HDAC4*	GCACAGTCCTTGGTTGGTG	AGAAACTGCTGATGCTGCTG
	*SIRT1*	GCCGACAACTTGTACGACGA	CACCGAACAGAAGGTTATCTCG
	*SIRT2*	CCCCCTCTTAACCAGCAGTT	GATGCCTGTTTAAGCCTTGG
	*SIRT3*	CTCAGCCTCTCCTCCAGAAA	TAATGCCTTCCCTGTCTCAG
	*SIRT6*	AGGGTGGGGCTTTTTGTA	CTCTGGGGTGTGGCTTCTT
	*SIRT7*	AGCAGAGCAGACACCATCCT	CAGCCCAGTCATCCTTCG
	*HDAC11*	GGGGGAGGGCAGAAGAAG	CCGCCTCACCAGTGTCTG
***Inflammatory markers***	*IL-10*	ACATCAGGGTGGCGACTCTA	AAGGTTTCTCAAGGGGCTGG
	*IL-6*	GTCCAGTTGCCTTCTCCCTG	AGCACGACCACGACCTTG
	*TNF-α*	TCTGGGCAGGTCTACTTTGG	GGTTGAGGGTGTCTGAAGGA
***Housekeeping gene***	*GAPDH*	AAGAAGGTGGTGAAGCAGGC	GTCAAAGGTGGAGGAGTGGG

**Table 2:** Clinical and biochemical characteristics of study subjects.

Clinical Parameters	NGT (*n* = 20)	T2DM (*n* = 20) ^a^	UI-DFU (*n* = 10) ^b^	I-DFU (*n* = 10) ^c^
Gender (M/F)	11M/9F	12M/8F	6M/4F	5M/5F
Age (years)	51.6 ± 1.3	51.5 ± 1.2	51.7 ± 1.2	51.5 ± 1.3
BMI (kg/m^2^)	26.1 ± 1.7	26.5 ± 1.1 ^ns^	27.9 ± 1.7 *	28.1± 1^ns^
SBP (mm Hg)	115.8 ± 4.2	125.30 ± 2.5 ****	131.7 ± 3.1 ****	135.4 ± 2.1 **
DBP (mm Hg)	74.6 ± 4.1	83.10 ± 1.8 ****	85.1 ± 2.77 ^ns^	88.6 ± 1.8 **
FPG (mg/dL)	90.5 ± 4.9	143.3 ± 11.6 ****	167.8 ± 14.5 ****	241.1 ± 14.5 ****
PPG (mg/dL)	108 ± 6.1	227.7 ± 4.7 ****	242.6 ± 5.3 ****	293.6 ± 27.6 ****
HbA1c (%)	5 ± 0.3	8.01 ± 0.49 ****	9.3 ± 0.5 ****	10.3 ± 0.5 **
TSC (mg/dL)	175.4 ± 10.1	182.5 ± 3.4 **	184.8 ± 7.8 ^ns^	187.1 ± 3.1 ^ns^
HDL-c (mg/dL)	49.1 ± 6.9	46.2 ± 5 ^ns^	44.7 ± 11.7 ^ns^	40.9 ± 8.7 ^ns^
LDL-c (mg/dL)	91.6 ± 6.6	104 ± 15 **	123.3 ± 3.9 ***	129 ± 6 *
Urea (mg/dL)	24.2 ± 3.2	29.1 ± 4.4 ***	32.6 ± 2.6 *	37.6 ± 1.4 ***
Creatinine (mg/dL)	0.7 ± 0.1	0.8 ± 0.2 *	0.9 ± 0.1 *	1 ± 0.1 ^ns^
HOMA-IR	1.0 ± 0.3	3.5 ± 0.9 ****	5.1 ± 0.7 ***	8 ± 0.6 ****
**Inflammation markers**				
CRP (mg/L)	2 ± 0.6	4 ± 0.8 ****	15.6 ± 2.5 ****	37.1 ± 6.2 ****
ESR (mm/hour)	2.9 ± 1.1	21 ± 2.1 ****	45.6 ± 3.3 ****	56.4 ± 3.6 ****
WBC (10^9^/L)	4.7 ± 1.1	6.8 ± 1.0 ****	8.1 ± 0.5 **	12.1 ± 0.6 ****

All data are reported as mean ± SD for continuous variables; **** *p* < 0.0001, *** *p* < 0.001, ** *p* < 0.01, * *p* < 0.05, ^ns^ insignificant; ^a^ indicates comparison made between NGT and T2DM; ^b^ indicates comparison made between T2DM and UI-DFU; ^c^ indicates comparison made between UI-DFU and I-DFU.

**Table 3:** Spearman’s correlation coefficient of *NRF2* with *HDACs* among DFU subjects.

*NRF2* vs. *HDACs*	r Value	*p* Value
*HDAC1*	−0.539	0.014
*HDAC2*	0.584	0.128
*HDAC3*	−0.446	0.048
*HDAC8*	0.036	0.933
*HDAC4*	−0.488	0.029
*HDAC11*	−0.302	0.114
*SIRT1*	0.628	0.003
*SIRT2*	0.155	0.598
*SIRT3*	−0.147	0.09
*SIRT6*	0.144	0.758
*SIRT7*	0.348	0.499

*p* and r values were calculated using the Spearman’s correlation test at 95% confidence intervals. Values in italics are statistically significant.

**Table 4:** Spearman’s correlation coefficient of *HDACs* with inflammatory markers among DFU subjects.

Variables	*IL-6*		*TNF-ɑ*		*IL-10*	
	r Value	*p* Value	r Value	*p* Value	r Value	*p* Value
*SIRTUIN1*	−0.683	0.042	−0.2	0.606	0.536	0.137
*HDAC1*	0.477	0.194	0.259	0.5	−0.424	0.255
*HDAC3*	0.383	0.308	0.6	0.088	−0.167	0.667
*HDAC4*	0.733	0.025	0.483	0.187	−0.561	0.116

*p* and r values were calculated using the Spearman’s correlation test at 95% confidence intervals. Values in italics are statistically significant.
